# Immediate Remote Cerebellar Hemorrhage and Seizure Following Revision Lumbosacral Fusion

**DOI:** 10.7759/cureus.1292

**Published:** 2017-05-30

**Authors:** Alvin Y Chan, Jeffrey P Mullin, Edward Benzel, William Bingaman

**Affiliations:** 1 Medical College of Wisconsin; 2 Neurosurgery, Cleveland Clinic

**Keywords:** general tonic-clonic seizures, durotomy

## Abstract

Cerebellar hemorrhage (CH) is a rare but devastating complication following spine surgery. It is associated with a compromise to dura integrity and typically has a delayed post-operative onset. Here, we describe a patient who suffered a CH that presented with a generalized tonic-clonic (GTC) seizure immediately after a revision lumbar fusion. The patient did not regain consciousness from anesthesia prior to the hemorrhage. There are no reports indicating that CHs can occur abruptly following a spine surgery. This case outlines the importance of remaining vigilant for signs of CH immediately after surgery.

## Introduction

Cerebellar hemorrhage (CH) is a rare but potentially devastating complication following spine surgery. Surgery in any part of the spine can cause CH, possibly due to dural damage [1-2]. A compromise in dura integrity (e.g., a tear or a duratomy) could result in cerebral spinal fluid (CSF) changes, which are associated with CHs [1, 3-4]. If a CH has occurred without a documented dural tear, other factors may be involved [5].

We describe the case of a 56-year-old male who underwent an L5-S1 interbody revision and suffered an immediate CH with a general tonic-clonic (GTC) seizure. It occurred right after the surgery where the dura was unintentionally opened. We believe this is one of the first cases where a patient suffered a CH prior to regaining consciousness following surgery. This highlights the need for vigilance of CH symptoms that occur immediately post-operatively.

## Case presentation

A 56-year-old male with a past surgical history of an L5-S1 interbody fusion presented with persistent back pain and L5 radiculopathy. The pre-operative imaging revealed that the interbody cage was displaced laterally with a right L5 foramen stenosis. He also had a pseudomeningocele formation that extended to the cross-connecting hardware. He opted for decompression and revision.

A prior durotomy was reopened to remove the previous cross-connecting hardware. It was closed primarily with a muscle patch sewn over. There was no CSF egress during the Valsalva maneuver. The rest of the surgery was uncomplicated. A subfascial Hemovac drain was placed and prophylactic vancomycin powder (1 gm) was used in the surgical wound. The estimated CSF leak and blood loss were 50 mL and 300 mL respectively. The patient remained hemodynamically stable throughout the procedure.

**Figure 1:**
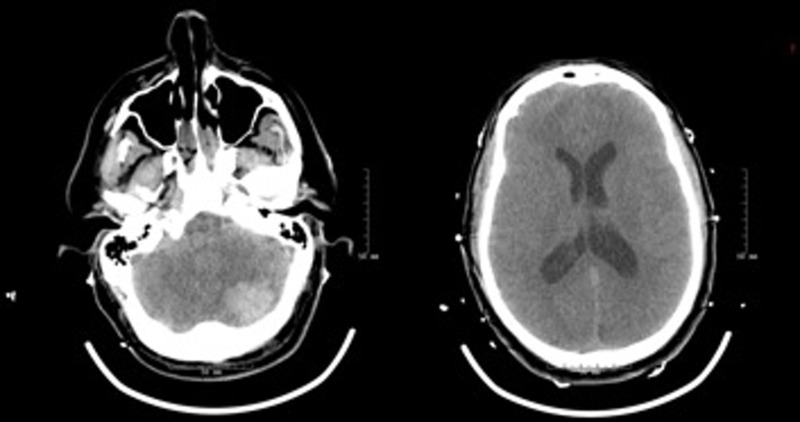
Interval computed tomography (CT) demonstrating enlarging cerebellar hemorrhage and dilated lateral ventricles

He suffered a GTC (30 minutes post-operatively) that was treated successfully with propofol and lorazepam. The computed tomography (CT) scan indicated the presence of a CH. He was transferred to the neurological intensive care unit (NICU) where he remained intubated. Sedation was withheld but he remained unconscious; both the pupils were round and reactive to light. He localized to stimulation with the upper extremities. A repeat CT scan three hours later showed an enlarging CH with dilated ventricles (Figure 1).

The Hemovac drain was removed due to concern for worsening hydrocephalus and an external ventricular drain (EVD) was placed. The opening pressure was 5 mmHg. The physical exam did not change even after CSF diversion. The patient improved over three days with medical management before extubation. He eventually returned to his neurological baseline. He had confusion and positional headaches during ambulation that resolved with lying supine. On post-operative day 7, the patient was taken to the operating room (OR) for exploration, which revealed a small CSF leak (from the previous durotomy) on repeated Valsalva maneuvers. A fibrin sealant spray was used and the wound was reclosed.

The patient was discharged at his neurological baseline 13 days after the original surgery. He has been seizure-free till date.

## Discussion

The report describes a patient with an immediate CH who presented with a seizure following an incidental durotomy that occurred during a revision lumbar fusion. CH after spine surgery typically has a delayed onset of hours to days [6]. Lee, et al. reported a case where a patient suffered a CH and seizure shortly after extubation [7]. Our case is unique because the CH occurred before the patient regained consciousness or was extubated, which adds to the literature on CH after spinal surgery. Although we cannot deny that the patient could have suffered a hypotensive episode that would explain the hemorrhage, this type of presentation is substantiated in literature and is most likely the best explanation for the presentation.

CH after spine surgery that presents with a GTC is a rare finding. Our review of the literature found four other documented cases. Two patients suffered CH and GTCs following a cervical spine surgery [8]. One patient presented following a sacral laminectomy that resulted in a CSF leak [9]. Another patient presented after an L3-L4 fusion [7]. A notable pattern is that CH is usually associated with GTCs rather than partial seizures. All patients including ours returned to their neurological baseline without a seizure recurrence.

The pathophysiology of seizures associated with CH is unknown. Bowers, et al. reasoned that subdural hemorrhages expose the brain to hemoglobin, which has proconvulsive effects [9]. We speculate that this may have been possible in our case. We also speculate that the vancomycin powder used in the surgical site could have been involved as a potential irritating agent. More research is required to understand the pathophysiology of seizures associated with CHs.

## Conclusions

The report describes how CH can occur without any delay following spine surgery. Surgeons should be vigilant for signs of CH immediately after the completion of surgery, especially if a durotomy has occurred. 
